# Postpartum intrauterine contraceptive device use and its associated factors in Ethiopia: systematic review and meta-analysis

**DOI:** 10.1186/s12978-021-01273-x

**Published:** 2021-11-13

**Authors:** Bekalu Getnet Kassa, Alemu Degu Ayele, Habtamu Gebrehana Belay, Adanech Getie Tefera, Gebrehiwot Ayalew Tiruneh, Netsanet Temesgen Ayenew, Gedefaye Nibret Mihiretie, Lebeza Alemu Tenaw, Abenezer Melkie Semahegn, Mulugeta Dilie Worku

**Affiliations:** 1 Department of Midwifery, College of Health Sciences, Debre Tabor University, Debre Tabor, Ethiopia; 2 Department of Anesthesia, College of Health Sciences, Debre Tabor University, Debre Tabor, Ethiopia; 3 School of Public Health, College of Health Sciences, Woldia University, Woldia, Ethiopia

**Keywords:** Postpartum, Utilization, Intrauterine device, Contraception, Ethiopia

## Abstract

**Background:**

The intrauterine contraceptive device, a type of long-acting reversible contraception, is one of the most effective and safe contraceptive methods. In Ethiopia, intrauterine contraceptive device is little known and practised to delay pregnancy. Therefore, this study aimed to assess post-partum intrauterine contraceptive device utilisation and its associated factors among women in Ethiopia.

**Method:**

In the current meta-analysis, variables were searched from different electronic database systems, including PubMed, Google Scholar, EMBASE, HINAR, Scopus, Web of Sciences, and Grey literature. Data were extracted using a standardised data collection measurement tool. The data were also analysed by using STATA 16 statistical software. *I*^2^ tests assessed heterogeneity between the studies. A random-effect model was used to forecast the pooled utilisation of postpartum intrauterine contraceptive device.

**Results:**

Twelve full-article studies were included. The pooled prevalence of post-partum intrauterine contraceptive device among women in Ethiopia was 21.63%. Occupation (OR = 4.44, 95% CI, 2.24–8.81), educational level of college and above (OR = 5.93, 95% CI, 2.55–13.8), antenatal care (OR = 2.09, 95% CI, 1.4–3.12), age (OR = 4.8, 95% CI, 2.3–10.04), good knowledge (OR = 4.16, 95% CI, 1.65–10.49), counseling (OR = 3.05, 95%CI, 1.41–6.63), husband support (OR = 11.48, 95% CI, 6.05–21.79) and awareness about IUCD (OR = 3.86, 95% CI, 1.46–10.2) were positively associated with utilization of postpartum intrauterine contraception device.

**Conclusions:**

Utilisation of post-partum intrauterine contraceptive device was significantly low. Scaling up women’s educational status and ANC use has paramount importance in increasing post-partum IUD use, which further improves maternal and child health in general. This finding may be useful in both reproductive health promotion at an individual level and policy-making regarding this issue.

**Supplementary Information:**

The online version contains supplementary material available at 10.1186/s12978-021-01273-x.

## Plain language summary

Family planning (FP) is widely recognised as a life-saving and health-improving intervention for women and children. An IUCD is a small, “T-shaped” intrauterine contraceptive device inserted into a woman’s uterus. It is also referred to as an IUD, a loop, or a coil. Post-partum IUCD is an intrauterine contraceptive device inserted during the post-partum period. Although several primary studies have been conducted in various regions of Ethiopia, there is no nationally representative evidence on the PPIUCD utilisation and the pooled effects of its determinants in Ethiopia. In this review, some of the factors associated with the post-partum intrauterine contraceptive device were pooled quantitatively, and some were not because of inconsistent classification (grouping) of the exposures concerning the outcome (post-partum intrauterine contraceptive device).

This systematic review and meta-analysis used the following electronic database; PubMed, Google Scholar, EMBASE, HINAR, Scopus, Web of Sciences, and Grey literature to search the primary articles. A total of 12 primary studies assessing the utilisation of postpartum intrauterine contraceptive device (PPIUCD) were included based on study eligibility criteria.

And also, in this study we found that the pooled prevalence of post-partum intrauterine contraceptive device among women in Ethiopia was 21.63%. Occupation, educational status, good knowledge, husband support, age, counselling, antenatal care follow-up, and awareness about IUCD were factors that affect the use of post-partum intrauterine contraceptive devices.

This systematic review and meta-analysis report that utilisation of post-partum intrauterine contraceptive device was significantly low. Therefore, scaling up women’s educational status, and ANC use has paramount importance in increasing post-partum IUD use, which further improves maternal and child health in general. Beside this health professional also should be give health education and promotion about the importance of PPIUCD.

**Supplementary Information:**

The online version contains supplementary material available at 10.1186/s12978-021-01273-x.

## Introduction

The global maternal mortality ratio is unacceptably high, with an estimated 42% of maternal deaths occurring during labour and delivery [1]. Every day, approximately 800 women die due to pregnancy or childbirth-related complications worldwide [2]. In 2015, developing regions were responsible for approximately 302,000 global maternal deaths, with Sub-Saharan Africa alone accounting for roughly 201,000, followed by Southern Asia (66,000) [3]. According to the EDHS 2016, Ethiopia has a high maternal mortality rate of 412 per 100,000 live births [4].

Family planning (FP) is widely recognised as a life-saving and health-improving intervention for women and children [5, 6]. Because of the woman's frequent contact with the health system over a relatively long period of time that makes an opportunity for counseling women about the adoption of family planning methods [7]. Closely spaced pregnancies within the first year post-partum are the riskiest for mother and child, resulting in an increased risk of adverse outcomes [8]. With this importance total contraceptive prevalence rate are 36%, but the prevalence rate of IUCD is still less than < 1% [4]. An IUCD (loop) is a small, “T-shaped” and made of flexible plastic with a thin copper wire coating contraceptive device inserted into a woman’s uterus. And also post-partum IUCD is a contraceptive device inserted during the post-partum period (up to 48 h after birth, ideally within 10 min of placenta delivery) [9]. The post placental IUCD insertion, the immediate post-partum IUCD insertion, and the trans-cesarean IUCD insertion are all appropriate times for IUCD insertion in the post-partum period [10–12]. Recent literature supports its safety, demonstrating low rates of infection, perforation, and expulsion that should not deter a clinician from offering it as a contraception option. Furthermore, IUCD insertion should be considered a viable option for the nulliparous and as an option during the post-partum period [12].

Post-partum IUD insertion is an ideal family planning methods for post-partum period to achieve the recommended pregnancy spacing. It is safe and effective up to 48 h insertion after placental delivery. However; PPIUCD insertion between 48 h upto 6 weeks post-partum period has been linked to a higher expulsion rate [13, 14].

Intrauterine device (IUD) insertion during the post-partum period is an ideal method for some women because it does not interfere with breastfeeding, is convenient for both women and their health care providers, and allows women to obtain safe, long-acting, highly effective contraception while they are already in the medical system [15, 16].

In Ethiopia, the utilisation of IUCD was low compared to other family planning methods [17]. According to the EDHS 2016, < 1% of modern family planning users use an immediate post-partum intrauterine contraceptive device [18]. However, in Ethiopia, the PPIUD is almost absent from the contraceptive method mix [19]. The Ethiopian Ministry of Health created a health sector transformation plan in 2015, intending to increase the contraceptive prevalence rate (CPR) to 55%. This would entail reaching an additional 6.2 million women and adolescent girls with family planning services by 2020, lowering total fertility to 3.0, and increasing utilisation of IUCD from 2 to 15% by 2020 [20].

Although several primary studies have been conducted in various regions of Ethiopia, there is no nationally representative evidence of the PPIUCD utilisation and the pooled effects of its determinants in Ethiopia. So, the goal of this study was to find the best available evidence of the pooled prevalence of PPIUCD utilisation and factors associated with its utilisation. This study’s findings will provide scientific evidence to program planners and policymakers for PPIUCD service improvement.

## Methods

### Study design and protocol

A systemic review and meta-analysis approach used to reviewed a relevant articles to estimate the pooled effect of PPIUCD utilisation and its associated factors among women in Ethiopia. This protocol was conducted following guidelines for the Preferred Reporting Items for Systematic Review and Meta-Analyses (PRISMA) [21].

### Eligibility criteria

#### Inclusion criteria

Study participants included those women of reproductive age, pregnant and post-partum, who resided in Ethiopia. This study included all published and unpublished observational (cross-sectional and case–control) studies on the utilisation of PPIUCD and factors affecting PPIUCD among women in Ethiopia. This review included studies done until February 12, 2020, and was written in the English language.

#### Exclusion criteria

Studies were excluded: (1) if the study was not published in English and we were unable to access a copy translated in the English language; (2) qualitative papers that did not include reproductive data for women of childbearing age to be included in the analysis; (3) cases studies, as most studies lacked robust quality reproductive data to include in the analysis and to reduce the potential for identifying a patient in the review; (4) secondary works (e.g., review articles, commentaries, editorials, or dissertations/thesis, conference abstracts that had not yet been published).

### Search strategy and data source

A literature search was performed by using PubMed, Google Scholar, EMBASE, HINAR, Scopus, Web of Sciences, and Grey literature. We also performed hand searched for cross-references to distinguish additional relevant articles. The PEO (Population, Exposure, and Outcomes) search format has used this review to search for pertinent studies.

#### Population

Women of reproductive age group, pregnant and post-partum women.

#### Exposure

Determinants of PPIUCD (socio-demographics such as age, educational status, occupational status); reproductive and family planning-related factors (history of FP, having ANC, knowledge about PPIUCD, ever heard about IUCD, Counseling about PPIUCD).

#### Outcome

Utilisation/use of PPIUCD among women.

Nine authors searched studies (BG, AD, HG, AG, GA, NT, GN, LA and AM) using comprehensive searching strategies. Initially, articles were searched by examining the full titles (“prevalence, utilisation of post-partum intrauterine contraceptive device and associated factors among women in Ethiopia”) and then keywords (“Prevalence “utilisation “use “uptake “post-partum intrauterine contraceptive device determinants” “predictors”, “associated factors “women, and “Ethiopia”). Besides this, studies were also searched from all included studies reference lists to find additional studies not included in our search strategies. Furthermore, to find relevant unpublished studies, Ethiopian universities’ digital libraries were searched (Addis Ababa University, University of Gondar, and Haramiya University). The searching periods were from January 12, 2020, to February 12, 2020 (Additional file 1).

### Identification and study selection

All identified studies were exported to the Endnote X7 reference manager software, and duplicated articles were excluded. Studies were screened after reading the title and abstracts. Eight authors (BG, AD, HG, AG, GA, NT and GN) screened and assessed articles independently. The studies full text was further assessed based on aims, methodology, participants/population, and critical findings (prevalence/utilisation of PPIUCD and factors affecting the use of PPIUCD). Any disagreements were resolved through discussion and consensus-based on established criteria or through an eighth, ninth and tenth investigator (LA, AM and MD) if consensus could not be reached.

### Quality assessment

Each study’s scientific strength and quality incorporated original observational (cross-section and case–control) study was assessed using the Newcastle–Ottawa Scale quality assessment tool adapted for cross-sectional study quality assessments [22]. The tool has three core components; the tool’s principal component is graded from five stars and mainly emphasises the methodological quality of each primary studies. The second components of the tool weigh up the equivalency of the primary studies included in this systematic review and meta-analysis. The tool’s last component assessed the quality of primary articles in statistical analysis and outcome point of view and was based on three stars. The qualities of each original study were weighted by nine authors independently using these pointers. Those primary studies with a medium score (satisfying 50% quality evaluation criteria) and high quality (≥ 7 out of 10) were enrolled for analysis. The nine investigators’ differences were managed by taking their quality evaluation outcomes (Additional file 2).

### Data extraction

After selecting the suitable studies selecting suitable studies, all necessary data were extracted by Seven authors (BG, AD, HG, AG, GA, NT and GN) independently using a pre-tested standardised data extraction form. This form includes primary author, year of publication, study setting, sample size, study design, response rate, the prevalence of PPIUCD, and specific factors associated with the use of PPIUCD. For the second objective (factors), the information extraction format was prepared for each specific factor, i.e., women’s age, educational status, ANC visit, awareness about IUCD, good knowledge about PPIUCD, counselled about PPIUCD, and husband/partner being supportive of IUCD use. In this study, variables were selected if two or more studies reported them as a significant factor. The last three authors resolved disagreements between the seven authors. If still there were disagreements between the ten authors, the consensus was reached by taking the mean score of the ten authors.

#### The outcome of interest

Prevalence of PPIUCD were the primary outcomes explored in this systematic review and meta-analysis. The second objective of the review was to determine the factors affecting the use of PPIUCD.

### Publication bias and heterogeneity

Rigorous searches (electronic/database search and manual search) have been used to minimise the risk of bias. For the valuation of the publication bias of the included studies, we used Funnel plots. The publication bias’s statistical significance was declared using the Egger regression test, setting *p* < 0.05. The Duval and Tweedie nonparametric trim and fill analysis using the random-effect analysis was conducted to account for publication bias for meta-analysis results, which showed the presence of publication bias (Egger test, *p* < 0.05) [23, 24]. Furthermore, to reduce the random variations among the primary study’s point estimates, subgroup analysis was conducted by study regions, study population and study design. Sensitivity analysis was also performed to identify the potential source of heterogeneity. Heterogeneity across studies was evaluated using inverse variance (*I*^2^) statistics with its corresponding p-value using the random-effect model.

### Statistical analysis

We used Microsoft Excel for data entry and STATA version 16 software for analysis. The associated factors of PPIUCD were examined based on eligibility criteria. We had considered at least two studies that reported at least one associated factor of PPIUCD in common with their measure of effect and 95% confidence interval (CI). The random-effects model based on the DerSimonian-Laird method was considered to assess variations between the studies. The results were presented using texts, tables, and forest plots with effect and 95% confidence interval measures. Statistical heterogeneity was tested via the *I*^2^ statistics at a *p-*value of ≤ 0.05 [25].

## Result

### Description of studies

**Fig. 1:**
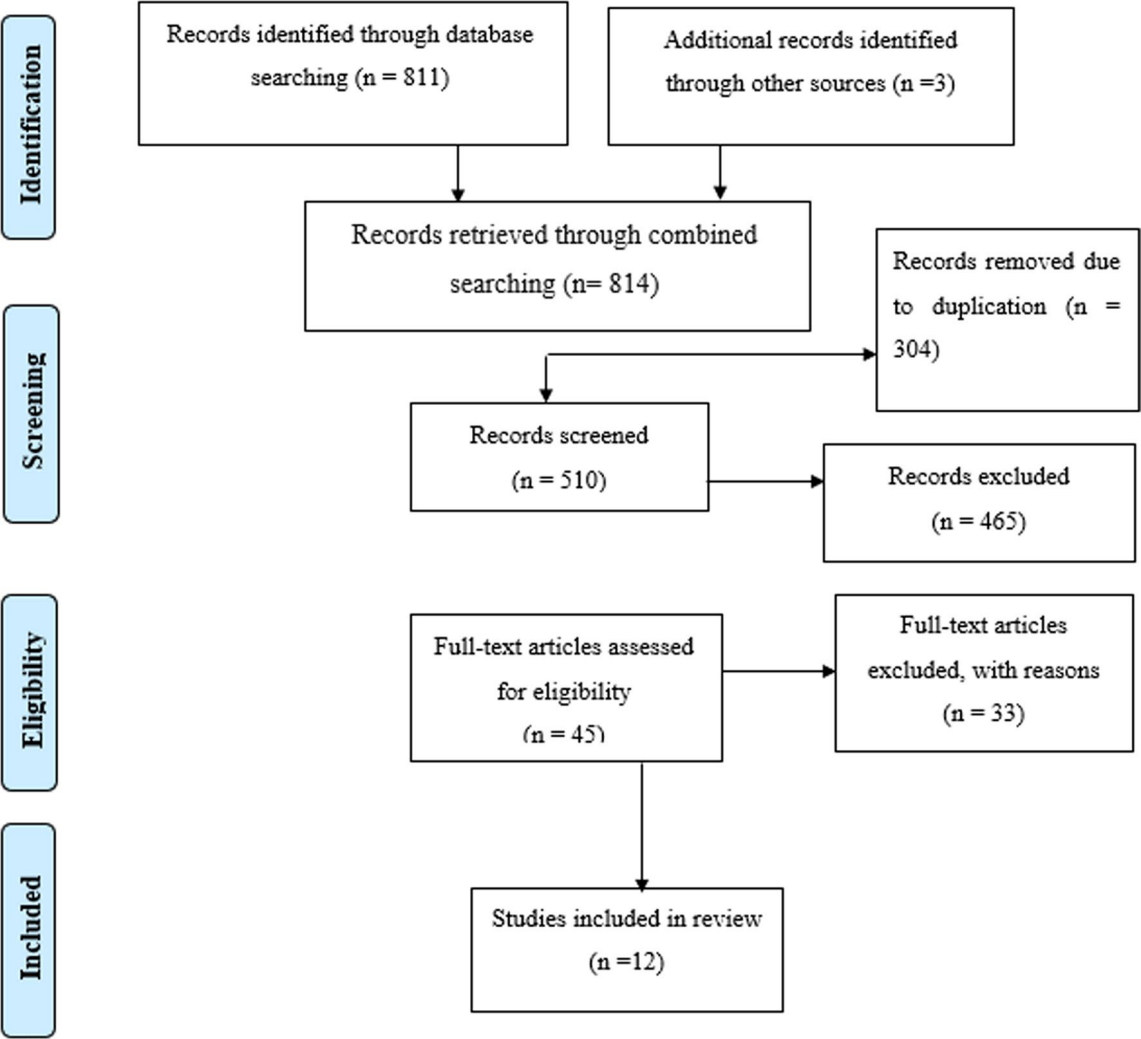
Flow chart describing the selection of studies for the systematic review and meta-analysis of the prevalence PPIUCD and associated factors among women in Ethiopia, 2021

A total of 814 primary studies were identified. From these 814 identified studies, 304 were excluded after reviewing their titles due to duplication, and 510 articles were further screened for potential inclusion. Out of these 465 articles, were excluded due to irrelevance, and 33 were removed due to inappropriate statistical analysis, irrelevant target population, inconsistent study report. Twelve articles fulfilled the inclusion criteria and were included in this systematic review and meta-analysis with a total population of 4367 women (Fig. 1).

### Characteristics of the included studies

These 12 eligible studies were observational study (cross-section and case–control) in design and reported in English. The sample size ranged from 188 women in Amhara [10] to 536 in South Nation Nationalities People of Region (SNNPR) [26]. The prevalence of PPIUCD reported was between 3.3% [10] and 35.2% [27] (Table 1). Once more, out of the twelve included studies, nine had a quality score of seven, and the remaining three had a quality score of eight. Hence, all of them had good quality (Additional file 2).

**Table 1:** Summary of the 12 observational studies included in the systematic review and meta-analysis assessing the prevalence of PPIUCD and its associated factors among women in Ethiopia, 2021

Authors	Publication year	Regions	Study design	Sample size	Response rate (%)	Prevalence
Geda et al.[24]	2021	Addis Ababa	Cross section	286	100	26.6
Ali et al.[23]	2016	Addis Ababa	Cross section	326	94.2	35.2
Sandy et al.[25]	2015	Addis Ababa	Cross section	336	100	7.7
Derege et al.[26]	2020	Addis Ababa	Case control	384	100	33.3
Animen et al.[27]	2018	Amhara	Cross section	241	100	13.3
Gonie et al.[28]	2018	Oromia	Cross section	465	92.3	12.4
Hagos et al.[6]	2020	Amhara	Cross section	188	97	3.3
Jemal M. et al.[22]	2020	SNNPR	Case control	536	95.1	34.3
Mandefro et al.[29]	2021	Amhara	Case control	450	93.3	33.3
Tefera et al.[30]	2017	SNNPR	Cross section	310	100	21.6
Melkie et al.[31]	2021	Amhara	Cross section	423	100	4.02
Daba [32]	2018	Oromia	Cross section	422	98.3	34.9

### Prevalence of postpartum intrauterine contraceptive device in Ethiopia

**Fig. 2:**
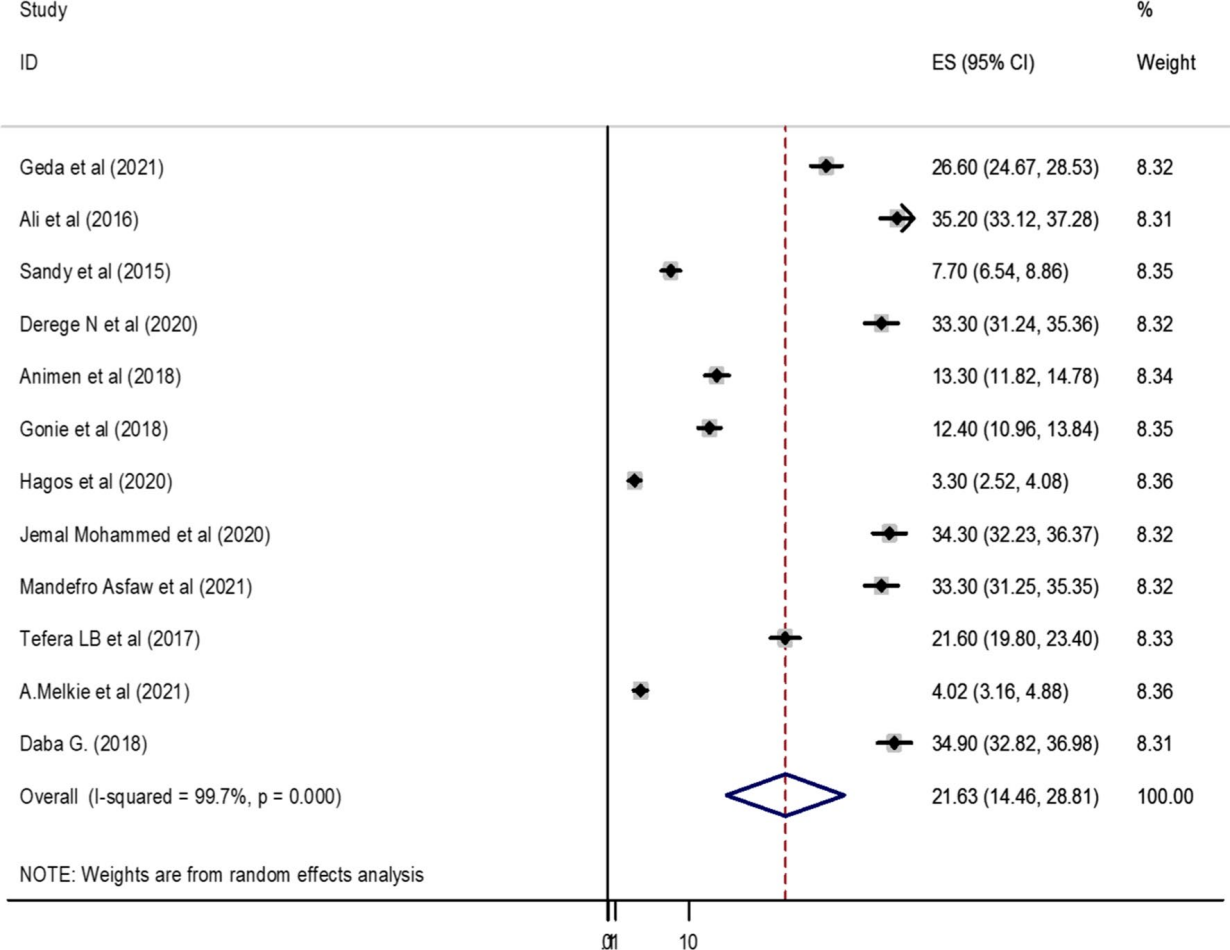
Forest plot of the pooled prevalence of postpartum intrauterine contraceptive device among women in Ethiopia, 2021

Based on the random effect model, the overall pooled prevalence of post-partum intrauterine contraceptive device among women in Ethiopia was 21.63% (95% CI: 14.46, 28.81) (Fig. 2).

### Publication bias and heterogeneity

**Fig. 3:**
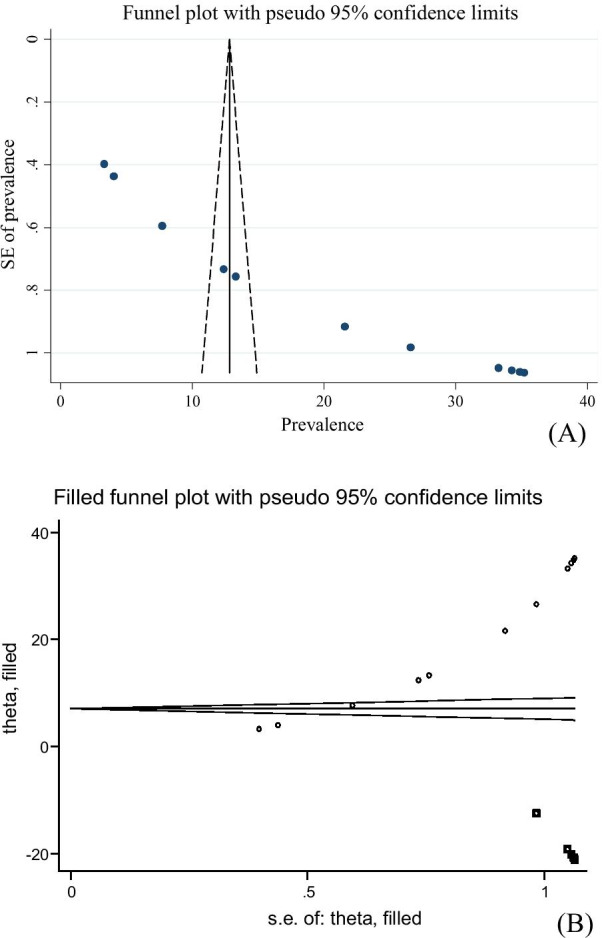
**A** Funnel plot to test publication bias of 12 studies. **B** Result of trim and fill analysis for adjusting publication bias of the 18 studies

Publication biases among the included studies were examined by using both funnel plots and Egger’s regression test. The results of funnel plots showed asymmetric shape, which indicates the presence of publication bias among those included studies (Fig. 3A). Objective assessments of publication bias by Egger’s regression test also showed the presence of publication bias across studies (*p*-value < 0.001). The Duval and Tweedie nonparametric trim and fill analysis were done to correct publication bias among the studies. Accordingly, publication bias was corrected when six missed studies were filled in the funnel plot by trim and fill analysis. After six studies were filled, a total of 18 studies were included and computed via the trim and fill analysis to produce the pooled prevalence of 8.177 (0.290, 16.065) by applying a random effect model (Fig. 3B).

According to the finding of this study, there was markedly high heterogeneity across the studies, as evidenced by *I*^2^ statistics (*I*^2^ = 99.7%, *P* ≤ 0.001). Due to this random effect model, meta-analysis was applicable to determine the pooled prevalence of post-partum intrauterine contraceptive device among women in Ethiopia.

### Sensitivity analysis

**Fig. 4:**
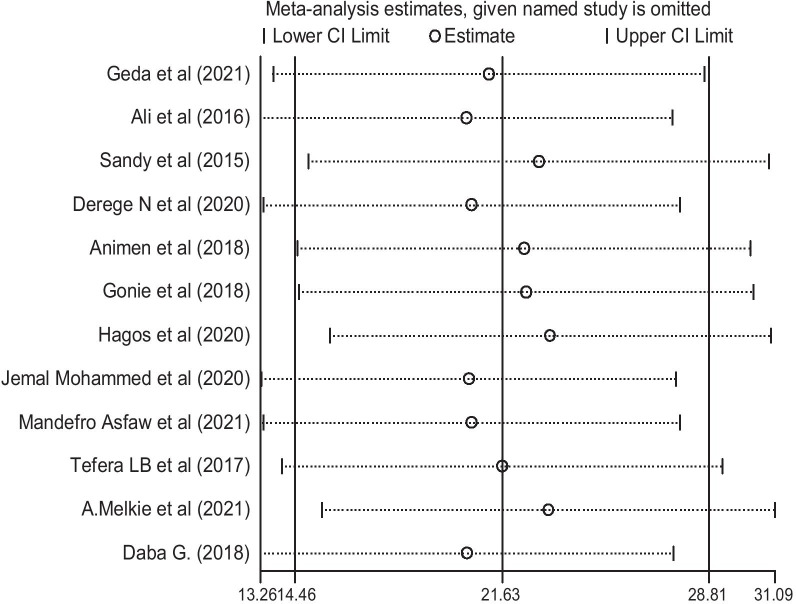
Sensitivity analysis of the use of PPIUCD among women in Ethiopia, 2021

In the current meta-analysis, to determine the potential source of heterogeneity seen in the pooled prevalence of post-partum intrauterine contraceptive device among women, the investigators performed a leave-one-out sensitivity analysis. The result of the sensitivity analysis indicated that the finding was not relaid on a particular study. The pooled prevalence of post-partum intrauterine contraceptive device was varied and ranged from 20.39% (13.26, 27.53%) to 23.31% (15.67, 30.93%) after deletion of six studies (Fig. 4).

### Subgroup analysis

In this meta-analysis, we computed subgroup analysis based on the study region. Thus, the highest prevalence was determined in South Nation Nationality and people region 27.94% (95% CI: 15.49, 40.38). We also executed subgroup analysis using the study population of the primary studies. The outcome of this subgroup analysis revealed that studies done with a study population of pregnant women 34.09 (95% CI: 32.52, 35.66) were higher in using PPIUCD compared to those categories of counterparts. Moreover, computed subgroup analysis with study design; that highest prevalence was determined in case–control study design 21.632(95% CI: 14.46, 28.81) (Table 2).

**Table 2:** Subgroup analysis of PPIUCD utilization among women in Ethiopia, 2021

Variables	Characteristics	Included studies	Number of study participants	Prevalence (95% CI)	I^2^(%), *P-value*
Region	Addis Ababa	4	1332	25.68 (11.073–40.296)	99.6, < 0.001
	Amhara	4	1302	13.43 (4.113–22.747)	99.6, < 0.001
	Oromia	2	887	23.64 (1.587–45.687)	99.7, < 0.001
	SNNPR	2	846	27.94 (15.493–40.385)	98.8, < 0.001
Study population	Postpartum women	5	1934	19.56 (8.609–30.516)	99.6, < 0.001
	Reproductive age women	5	1627	18.73 (7.464–29.992)	99.9, < 0.001
	Pregnant women	2	806	34.09 (32.524–35.660)	13.1, 0.283
Study design	Case–control	3	1370	21.632 (14.457–28.807)	0.00, = 0.741
	Cross-section	9	2997	17.633 (10.635–24.630)	99.6, < 0.001

### Associated factors of the post-partum intrauterine contraceptive device in Ethiopia

In this review, some of the factors associated with the post-partum intrauterine contraceptive device were pooled quantitatively, and some were not because of inconsistent classification (grouping) of the exposures concerning the outcome (post-partum intrauterine contraceptive device).

**Fig. 5:**
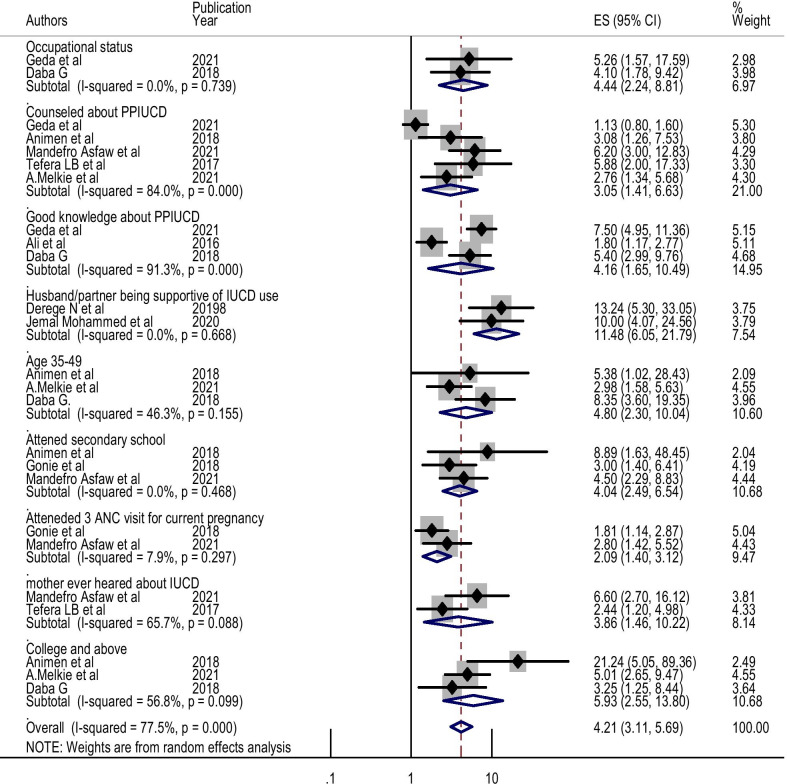
Factors associated with the use of PPIUCD among women in Ethiopia, 2021

Two studies reviewed indicated that the pooled effect of occupational status of housewife were nearly three times more likely to utilise post-partum intrauterine contraceptive devices compared to those women who have no occupational status (AOR = 4.44, 95% CI: 2.24, 8.81) (Fig. 5).

Also, there was an association between good knowledge about PPIUCD and the use of post-partum intrauterine contraceptive device as described in the three studies. The pooled analysis demonstrated a statistical difference among them (AOR = 4.16, 95% CI: 1.65, 10.49) (Fig. 5).

Five studies described PPIUCD; one study revealed no association with counselled about PPIUCD, while in four studies, counselling about PPIUCD was found to affect the use of the post-partum intrauterine contraceptive device significantly. The overall pooled estimate indicated that counselled about PPIUCD significantly associated with a post-partum intrauterine contraceptive device (AOR = 3.05, 95% CI: 1.41, 6.63) (Fig. 5).

Two studies presented the husband/partner being supportive of IUCD use with PPIUCD utilisation; all of them were indicated strongly associated with utilisation of PPIUCD. Our pooled estimates showed that there was an association with the use of PPIUCD (AOR = 11.48, 95% CI: 6.05, 21.79) (Fig. 5).

Six studies described the educational status of participant mothers; all of them were found to affect the use of PPIUCD significantly. The overall pooled estimate indicated that educational level of secondary school,college and above was significantly associated with the use of PPIUCD (AOR = 4.04, 95% CI: 2.49, 6.54) and (AOR = 5.93, 95% CI: 2.55, 13.80) respectively (Fig. 5).

Two studies also described have a history of ANC visits for the current pregnancy had associated with the use of PPIUCD. The pooled estimate indicated that attended three times ANC visits for the current pregnancy/index/ had a positive effect on the use of PPIUCD (AOR = 2.09, 95% CI: 1.40, 3.12) (Fig. 5).

Mother who have an awareness about IUCD was another factor that affects the use of PPIUCD. This is supported by the pooled estimate of two independent studies (AOR = 3.86, 95% CI: 1.46, 10.22) (Fig. 5).

Two studies presented the mother’s age with PPIUCD; all of them indicated that women in the age group of between 35 and 49 years had an association with the use of PPIUCD. Our pooled estimates showed an association with this age (AOR = 4.80, 95% CI: 2.30, 10.04) and the use of PPIUCD in Ethiopia (Fig. 5).

## Discussion

This systematic review and meta-analysis reviewed indicated that the pooled prevalence of PPIUCD utilisation among women in Ethiopia was 21.63% ranging from 14.46 to 28.81%. Even though no comparable meta-analysis study was conducted in this specific research question, the finding was similar with a study done in Uganda (16.3%) [28]. However, this review and meta-analysis finding was lower than a systematic review and meta-analysis conducted in sub-Saharan Africa (41.2%) [29], and comparative study of follow-up outcomes in India (58.3 vs 41.7%) inserted intra-cesarean and after vaginal delivery, respectively [30]. The discrepancy may be due to cultural influences and the relatively high unmet need for family planning in Ethiopia [31].

In the present review and meta-analysis, subgroup analysis was done by regions, study population, and study design indicated that there were variations in the utilisation of PPIUCD among different included regions, population and study design in Ethiopia. Accordingly, the highest in utilization of PPIUCD was observed in Addis Ababa 25.68% (95% CI: 11.07, 40.29) [27, 32–34], 34.09% (95% CI: 32.52, 35.66) [32, 35], and 21.63% (95% CI: 14.45, 28.81) [26, 32, 36] by region, study population and study design respectively. The observed differences could be due to culture, residence, educational status and religious differences across the region. Furthermore, it could be due to differences in access to health care facilities and information on the efficacy of contraception during the post-partum period.

In the current meta-analysis, the mother’s occupational status was positively associated with the utilisation of PPIUCD. Women who work as housewives were 4.44(95% CI: 2.24, 8.81) times more likely to utilise PPIUCD than those who work gov’t employment. This is supported by a primary study conducted in Uganda [28]. This may be due to women who has a housewife is high risk for repeated pregnancy and have more children; to avoid such condition; better use PPIUCD than other counterparts.

The present review and meta-analysis also demonstrated that good knowledge about PPIUCD has significantly associated with the utilisation of PPIUCD 4.16 (95% CI: 1.65, 10.49) compared to their counterparts. This is similar with the study conducted in India [37]. It also revealed that counselled about PPIUCD were strongly influence the use of PPIUCD. Women who had counselled about PPIUCD were 3.05 (95% CI: 1.41, 6.63) more likely to utilise than those who had not counselled about PPIUCD. Furthermore, awareness about IUCD were 3.86 (95% CI: 1.46, 10.22) more likely to utilise PPIUCD than those counterparts. This is consistent with a study done in Uganda, Tanzania, Sri Lanka, India and Nepal [28, 38]. The possible justification could be that counselling may allow women to get accurate information about PPIUCD that can change their attitudes and behaviours by avoiding rumours and misconceptions, which may hinder the acceptance of post-partum intrauterine contraceptive devices.

In this meta-analysis, women whose age was greater than 35 years of age were 4.8 times more likely to utilise PPIUCD than younger women. Similarly, this evidence is supported by previous studies conducted in Tanzania, Sri Lanka and Nepal [38]. This may be because mothers who have fewer alive children rarely use contraception as they need more children. On the other side, these findings were opposite the study done in India [33]; older women were less likely to accept and utilise PPIUCD.

The current meta-analysis also revealed that husband/partner supportive of IUCD use strongly influenced PPIUCD. Women who had husband/partner supportive of IUCD use were 11.48 (95% CI: 6.05, 21.79) more likely to utilise than those who had no support from their families. This could be because, in Ethiopia, women are typically dependent on their husband’s decisions, despite efforts by the government and various nongovernmental organisations to empower women. Furthermore, it may be due to the desire to protect their marriages and families from unresolved conflicts and avoid divorce. Another way to put it is that decisions made jointly with the agreement of both couples will have a better outcome because family planning is not just one partner’s concern.

The educational status of the mother was positively associated with the utilisation of PPIUCD. Women who have education attend secondary and above were 4.04 (95% CI: 2.49, 6.54) times more likely to utilise PPIUCD than those counterparts. This finding is similar with a study done in Uganda [28] and India [39, 40]. Hence, those women with higher education levels might improve women’s knowledge on contraception use. Furthermore, this finding suggests that education has a positive impact on women’s willingness to use PPIUCD.

Moreover, we found a significant association between women who have ANC visits with the utilisation of PPIUCD. A woman who have antenatal visits were more likely to utilise PPIUCD when compared to those counterparts. This is supported by a study conducted in the same manner in India [41, 42] and Egypt [43], which confirms that counselling during the antenatal period was a critical factor in increasing the acceptance of PPIUCD. Women who have ANC visits may have accepted PPIUCD because healthcare workers counselled them during their ANC visits. It could also be explained that during ANC visits, health care providers cleared up misconceptions about the use of PPIUCD. As a result, providing effective contraceptive counselling during ANC visits could address any misconceptions and encourage women to accept PPIUCD use immediately following delivery.

### Strength and limitations of the study

This review searched not only published but also unpublished articles using various databases. However, this review is not without limitations because the primary studies included in this review were limited to some areas of the country, and other regions may be underrepresented. This review considered only articles published in the English language, which may have resulted in the omission of studies that could have been published in other languages. Furthermore, the review included only a few primary studies with small sample sizes, which could impact the estimated magnitude of PPIUCD use.

## Conclusions

The utilisation of post-partum intrauterine contraceptive device was significantly low. Occupation, educational status, good knowledge about PPIUCD, husband/partner support, age, counselling, antenatal care follow-up and awareness about IUCD were factors shown to affect the use of PPIUCD. Therefore; encouraging women's education and informing health professionals to give health education for the importance of PPIUCD may enhance PPIUCD utilization. And also; strengthening adherence to focused ANC utilisation should be given due attention to encourage women in utilising contraception during the postnatal period; which further improves maternal and child health in general. This finding may be useful in both reproductive health promotion at an individual level and policy-making regarding this issue.

## Supplementary Information

**Additional file 1. **Searching strategy for Postpartum intrauterine contraceptive device and associated factors in Ethiopia 2021.

**Additional file 2. **Newcastle-Ottawa Quality Assessment Scale for cross-sectional studies to assess the use of PPIUCD among women in Ethiopia, 2020.

## Data Availability

The data that support the review findings of this study are available upon a reasonable request to the corresponding author.

## Abbreviations

ANC: Antenatal Care
AOR: Adjusted Odd Ratio
CI: Confidence Interval
EDHS: Ethiopia Demographic and Health Survey
FP: Family Planning
IUD: Intrauterine Device
IUCD: Intrauterine Contraceptive Device
PPIUCD: Postpartum Intrauterine Contraceptive Device
SNNPR: South Nation Nationality People Region
