# Supplementary material for: Postpartum intrauterine contraceptive device use and its associated factors in Ethiopia: systematic review and meta-analysis
Source: Reprod Health. 2021 Nov 13;18:225. doi: 10.1186/s12978-021-01273-x (PMC8590214; doi:10.1186/s12978-021-01273-x)
Supplement: Supplementary file 1 — Additional file 1. Searching strategy for Postpartum intrauterine contraceptive device and associated factors in Ethiopia 2021. [file 12978_2021_1273_MOESM1_ESM.docx]

**Additional file 1:** Searching strategy for Postpartum intrauterine contraceptive device and associated factors in Ethiopia 2021.

| Databases | Searching terms | Number of studies |
| --- | --- | --- |
| PubMed | ("Women"[MeSH Terms] OR "Women"[All Fields]) AND "Postpartum period"[MeSH Terms] OR ("postpartum"[All Fields] AND "period"[All Fields]) OR "postpartum period"[All Fields] OR "postpartum"[All Fields] AND ("intrauterine devices"[MeSH Terms] OR ("intrauterine"[All Fields] AND "devices"[All Fields]) OR "intrauterine devices"[All Fields] OR ("intrauterine"[All Fields] AND "contraceptive"[All Fields] AND "device"[All Fields]) OR "intrauterine contraceptive device"[All Fields]) AND Associated[All Fields] AND Factors[All Fields] AND ("Ethiopia"[MeSH Terms] OR "Ethiopia"[All Fields]) | 266 |
| Google scholar | "Utilization" AND "Postpartum" AND "Contraception" AND "Intrauterine Device" AND "Magnitude" OR "Prevalence" AND "Factors" OR "Predictors" OR "Determinant" OR "Influencing" AND "Ethiopia". | 407 |
| HINARI | "Utilization" AND "Postpartum" AND "Contraception" AND "Intrauterine Device" AND "Magnitude" OR "Prevalence" AND "Factors" OR "Predictors" OR "Determinant" OR "Influencing" AND "Ethiopia". | 37 |
| Others databases |  | 104 |
| Total retrieved |  | 814 |
| Included |  | 12 |
