# Supplementary material for: Postpartum intrauterine contraceptive device use and its associated factors in Ethiopia: systematic review and meta-analysis
Source: Reprod Health. 2021 Nov 13;18:225. doi: 10.1186/s12978-021-01273-x (PMC8590214; doi:10.1186/s12978-021-01273-x)
Supplement: Supplementary file 2 — Additional file 2. Newcastle-Ottawa Quality Assessment Scale for cross-sectional studies to assess the use of PPIUCD among women in Ethiopia, 2020. [file 12978_2021_1273_MOESM2_ESM.docx]

**Additional file 2:** Newcastle-Ottawa Quality Assessment Scale for cross-sectional studies to assess the use of PPIUCD among women in Ethiopia, 2020.

| Authors | Representatives | Sample size | Non -responders | Ascertainment | comparability | outcome | Quality score |
| --- | --- | --- | --- | --- | --- | --- | --- |
| Geda et al (2021) | 1 | 2 | 1 | 1 | 1 | 1 | 7 |
| Ali et al (2016) | 1 | 2 | 1 | 2 | 1 | 1 | 8 |
| Sandy et al (2015) | 1 | 1 | 1 | 2 | 1 | 1 | 7 |
| Derege et al (2020) | 1 | 1 | 1 | 2 | 1 | 1 | 7 |
| Animen et al (2018) | 1 | 1 | 2 | 2 | 1 | 1 | 8 |
| Gonie et al (2018) | 1 | 2 | 1 | 1 | 1 | 1 | 7 |
| Hagos et al (2020) | 2 | 1 | 1 | 1 | 1 | 1 | 7 |
| Jemal M. et al (2020) | 1 | 1 | 1 | 1 | 2 | 1 | 7 |
| Mandefro Asfaw et al (2021) | 1 | 1 | 1 | 1 | 2 | 1 | 7 |
| Tefera LB et al (2017) | 1 | 2 | 1 | 1 | 2 | 1 | 8 |
| A. Melkie et al. (2021) | 2 | 1 | 1 | 1 | 1 | 1 | 7 |

**Interpretation of the score**: Very Good Studies: 9-10 points, Good Studies: 7-8 points, Satisfactory Studies: 5-6 points and Unsatisfactory Studies: 0 to 4 points
